# Introgression of an adult-plant powdery mildew resistance gene *Pm4VL* from *Dasypyrum villosum* chromosome 4V into bread wheat

**DOI:** 10.3389/fpls.2024.1401525

**Published:** 2024-06-20

**Authors:** Yi Wei, Ting Zhang, Yinyu Jin, Wen Li, Lingna Kong, Xiaoxue Liu, Liping Xing, Aizhong Cao, Ruiqi Zhang

**Affiliations:** ^1^ College of Agronomy of Nanjing Agricultural University, State Key Laboratory of Crop Genetics and Germplasm Enhancement and Application, JCIC-MCP, Nanjing, China; ^2^ Zhongshan Biological Breeding Laboratory, Nanjing, Jiangsu, China

**Keywords:** wheat, powdery mildew, *Dasypyrum villosum*, *Pm4VL*, T7DL· 7DS-4V#6L translocation

## Abstract

Powdery mildew caused by *Blumeria graminis* f. sp. tritici (*Bgt*) seriously threatens wheat production worldwide. It is imperative to identify novel resistance genes from wheat and its wild relatives to control this disease by host resistance. Dasypyrum villosum (2n = 2x = 14, VV) is a relative of wheat and harbors novel genes for resistance against multi-fungal diseases. In the present study, we developed a complete set of new wheat-*D. villosum* disomic introgression lines through genomic *in situ* hybridization (GISH), fluorescence *in situ* hybridization (FISH) and molecular markers analysis, including four disomic substitution lines (2n=42) containing respectively chromosomes 1V#6, 2V#6, 3V#6, and 6V#6, and four disomic addition lines (2n=44) containing respectively chromosomes 4V#6, 5V#6, 6V#6 and 7V#6. These lines were subsequently evaluated for their responses to a mixture *Bgt* isolates at both seedling and adult-plant stages. Results showed that introgression lines containing chromosomes 3V#6, 5V#6, and 6V#6 exhibited resistance at both seedling and adult-plant stages, whereas the chromosome 4V#6 disomic addition line NAU4V#6-1 exhibited a high level of adult plant resistance to powdery mildew. Moreover, two translocation lines were further developed from the progenies of NAU4V#6-1 and the Ph1b mutation line NAU0686-ph1b. They were T4DL·4V#6S whole-arm translocation line NAU4V#6-2 and T7DL·7DS-4V#6L small-fragment translocation line NAU4V#6-3. Powdery mildew tests of the two lines confirmed the presence of an adult-plant powdery mildew resistance gene, Pm4VL, located on the terminal segment of chromosome arm 4V#6L (FL 0.6-1.00). In comparison with the recurrent parent NAU0686 plants, the T7DL·7DS-4V#6L translocation line NAU4V#6-3 showed no obvious negative effect on yield-related traits, providing a new germplasm in breeding for resistance.

## Introduction

Bread wheat (*Triticum aestivum* L., 2*n* = 6*x* = 42, AABBDD) provides approximately 20% of the calories required by humankind (Liu et al., 2022). However, some diseases seriously threaten wheat production. Powdery mildew (caused by *Blumeria graminis* f. sp. *tritici*, *Bgt*), a wheat major disease in temperate regions, can cause epidemics, resulting in yield losses of up to 70% in susceptible cultivars (Gao et al., 2018). Thus, it is imperative to identify resistance genes from wheat and its wild relatives to control this disease by developing and planting resistant varieties.

Plants have evolved various types of resistance during the long-term coevolution with pathogens. Many host plants have resistance to pathogens at all stages (ASR). Such resistance genes typically encode nucleotide-binding leucine-rich repeat (NLR) receptors, recognizing specific pathogen avirulence (Avr) proteins and providing race-specific resistance (Develey-Rivière and Galiana, 2007). In other cases, the host plant is restricted to adult plant resistance (APR). Some APR genes encode non-NLR receptors and confer race non-specific durable resistance (Whalen, 2005). To date, more than 70 powdery mildew resistance (*Pm*) genes have been explored in wheat and its wild relatives (McIntosh et al., 2020; Li et al., 2023). However, few of them are APR genes. In addition, 18 *Pm* genes have been cloned and characterized so far. Most of the isolated *Pm* genes encode coiled-coil nucleotide-binding leucine-rich repeat (CNL) receptors that recognize specific pathogen avirulence (Avr) proteins, whereas the two adult-plant resistance genes *Pm38* and *Pm46* encode an ATP-binding cassette (ABC) transporter and a hexose transporter involved in sugar uptake, respectively (Moore et al., 2015; Krattinger et al., 2019). Therefore, exploring novel APR genes contribute to not only breeding durable resistance but also dissecting molecular mechanisms of plant immunity.

Many disease resistance genes were introgressed from wheat wild relatives, enriching the modern wheat genetic diversity (Friebe et al., 1996), such as *Pm8/Sr31/Yr9/Lr26* genes introgressed from rye by T1BL·1RS translocation (Hurni et al., 2013), and *Lr19* introgressed from *Lophopyrum elongatum* by T7DL·7Ag translocation (Gupta et al., 2006). Notably, six powdery mildew resistance genes have been introgressed from *Dasypyrum villosum* L. (2*n* = 2*x* = 14, VV), an annual related species of wheat native to a northeastern part of the Mediterranean region and Caucasus area. Of these genes, *Pm21*, *Pm5V*, and *Pm3VS* were ASR genes (Xing et al., 2018; Zhang et al., 2022; Hou et al., 2024), *Pm55* and *Pm67* showed development-stage and tissue-specific resistance (Zhang et al., 2016, 2021), and *Pm62* was an APR gene (Zhang et al., 2018). Gene cloning showed that *Pm21*, *Pm55*, and *Pm5V* encode CNL protein receptors, and *Pm55* and *Pm5V* were allelic and renamed as *Pm55a* and *Pm55b*, respectively (Lu et al., 2024). However, *Pm55a* and *Pm55b* interacted differently with a linked inhibitor gene, *SuPm55*, to cause different resistance to wheat powdery mildew.

As an outcrossing diploid species, *D. villosum* resulted in complex haplotypes within the genetic locus of interest. At least six *D. villosum* accessions (V#1 to V#6) so far were used to develop addition, substitution, or translocation lines, and several disease resistance genes were located on specific chromosome arms (De Pace et al., 2011; Książczyk et al., 2011). *Sr52*, a stem rust resistance gene, was identified on Sicilian *D. villosum* accession (V#2) chromosome arm 6V#2S (Qi et al., 2011). Two powdery mildew resistance genes *Pm21* and *Pm55* were respectively identified on the chromosome arms 6V#4S and 5V#4S of *D. villosum* accession GP005 (V#4) (Zhang et al., 2016; Xing et al., 2018). The use of inferior genotypes, such as CS, as initial recipients of alien introgressions hinders the utilization of introgressed genes in breeding programs. Commercial cultivar NAU0686 was used to develop a novel set of introgression lines from the fifth *D. villosum* accession 01I140 (V#5), and four resistance genes *Pm5V*, *Yr5V*, *Pm62*, and *Pm67* were identified on the chromosome arms 5V#5S, 2V#5L, and 1V#5S, respectively (Zhang et al., 2018, 2021, 2022). Although the sixth *D. villosum* accession 01I139 (V#6) was also crossed with NAU0686, only chromosome 3V#6 with a powdery mildew resistance gene *Pm3VS* was introgressed into bread wheat so far (Hou et al., 2024).

Here, we developed and characterized a set of wheat–*D. villosum* accession 01I139 introgression lines in bread wheat cv NAU0686 background, including four disomic substitution lines, DS1V#6 (1D), DS2V#6 (2D), DS3V#6 (3D), and DS6V#6 (6D), and four disomic addition lines, DA4V#6, DA5V#6, DA6V#6, and DA6V#6. All the introgression lines were evaluated for their responses to a mixture of *Bgt* isolates at both seedling and adult-plant stages. A novel adult-plant powdery mildew resistance gene *Pm4VL* was identified on the long arm of chromosome 4V#6. Furthermore, a wheat–*D. villosum* T7DL·7DS-4V#6L translocation line with gene *Pm4VL* was developed, which could be a useful germplasm resource for wheat breeding.

## Materials and methods

### Plant materials

A durum–*D. villosum* amphiploid line AABBVV-3 (2*n* = 6*x* = 42, AABBVV) was developed from the crossing between *D. villosum* accession 01I139 (#6) and durum wheat ZY1286 (2*n* = 4*x* = 28, AABB, introduced from CIMMYT) (Hou et al., 2024). Spring bread wheat cv NAU0686 (highly susceptible to powdery mildew) was used to cross with AABBVV-3. To accelerate the alien introgression process, the progenies of NAU0686/AABBVV-3 were planted in a custom-made growth chamber (3 m × 3 m × 3 m) illuminated with LED lights set to an 18-h photoperiod (6 h of darkness) and temperatures of 22°C and 18°C. From the progenies of BC_2_F_2_ and BC_2_F_3_ of AABBVV-3/2*NAU0686, eight disomic lines were identified. A locally adapted ph1b-carrying line NAU0686-ph1b, deriving from a BC_4_ plant obtained by backcrossing the Chinese Spring *Ph1b* deletion line CS-ph1b to NAU0686, was used to cross with the DA4V#6 line to induce the alien chromosome translocation. Their F_2_ individuals were genotyped by PCR markers, 4VL-24 and 4VS-99, to ensure that they had retained the chromosome 4V#6 in a ph1bph1b background. The PCR primers are listed in **Supplementary Table S1**, following the methods described by Zhang et al. (2022). All the genetic resources used and developed in the present study are listed in **Table 1** and are maintained at the Cytogenetics Institute, Nanjing Agricultural University (CINAU).

**Table 1:** Summary of the genetic stocks developed and used in this study.

Line	Chromosome structure	Description
NAU0686	AABBDD (2n=42)	High-yield wheat cultivar highly susceptible to powdery mildew, used as the recurrent parent for all alien introgression lines
NAU0686-ph1b	AABBDD (2n=42)	The *Ph1* gene mutation line, pedigree: NAU0686/CS-ph1b//4*NAU0686
01I139	V#6V#6 (2n=14)	*Dasypyrum villosum* collected from Greece
ZY1286	AABB (2n=28)	Spring durum wheat used to cross with *Dasypyrum villosum* 01I139
AABBVV-3	AABBVV (2n=42)	*Durum* 1286- *D. villosum* 01I139 amphiploid
NAU1V#6–1	DS1V(1D) (2n=42)	Chromosome 1V#6 of 01I139 substituted chromosome 1D of NAU0686
NAU2V#6–1	DS2V(2D) (2n=42)	Chromosome 2V#6 of 01I139 substituted chromosome 2D of NAU0686
NAU3V#6–1	DS3V(3D) (2n=42)	Chromosome 3V#6 of 01I139 substituted chromosome 3D of NAU0686
NAU4V#6–1	DA4V (2n=44)	NAU0686 adding a pair of chromosome 4V#6 of 01I139
NAU4V#6–2	T4DL·4VS	Compensation translocation line in which chromosome arm 4VS of 01I139 substituted 4DS of NAU0686
NAU4V#6–3	T7DL·7DS-4VL	Small-segment translocation line in which a terminal fragment of 4V#6L of 01I139 substituted a fragment of 7DS of NAU0686
NAU5V#6–1	DA5V (2n=44)	NAU0686 adding a pair of chromosome 5V#6 of 01I139
NAU6V#6–1	DA6V(6D) (2n=44)	NAU0686 adding a pair of chromosome 6V#6 of 01I139
NAU6V#6–2	DS6V(6D) (2n=42)	Chromosome 6V#6 of 01I139 substituted chromosome 6D of NAU0686
NAU7V#6–1	DA7V(7D) (2n=44)	NAU0686 adding a pair of chromosome 7V#6 of 01I139

### Cytogenetic analysis

A platform described by Zhang et al. (2022) was used to accelerate alien gene introgression into bread wheat by combining chromosome engineering and speed breeding. *D. villosum* chromosomes were detected by the combination of genomic *in situ* hybridization (GISH) and fluorescence *in situ* hybridization (FISH) techniques. The mitotic metaphase chromosome spreads were obtained from the seedling root-tip cells soaked in cetyltrimethylammonium bromide (0.2 μmol/L) solution for 2 h at 24°C (King et al., 2017). *D. villosum* 01I139 genome DNA, labeled with fluorescein-12-dUTP and showing green signal, was used as a probe for GISH. The oligonucleotide probes Oligo-pAs1–2,5 and (GAA)_10_, labeled with 6-carboxytetrmethylrhodamine (TAMRA, red signal) and 6-carboxyfluorescein (6-FAM, green signal), respectively, and synthesized by Shanghai Sangon Biotech Co., Ltd. (Shanghai, China), were used to respectively identify the wheat D and B genome chromosomes by nondenaturing FISH, following the procedures described by Du et al. (2017). To complete the process, the chromosomes on the slides were counterstained with 4,6-diamidino-2-phenylindole (DAPI) (Invitrogen Life Science, Carlsbad, CA, USA). All cytological images were observed under an Olympus BX60 microscope (Olympus Co., Tokyo) and captured with a SPOT Cooled Color Digital Camera (Diagnostic Instruments, Sterling Heights, MI, USA).

### Molecular marker analysis

DNA was extracted from young leaves of materials using the cetyl trimethyl ammonium bromide (CTAB) method. Sixteen previously reported IT molecular markers specific to 1V–7V chromosome arms listed in **Supplementary Table S1** were used to screen for the introgression lines from *D. villosum* 01I139 (Zhang et al., 2017a). PCR amplification was carried out in an iCycler thermalcycler (Bio-Rad Laboratories, Emeryville, Calif., USA) in a 10-μL reaction, containing 40 ng of genomic DNA, 2.0 μmol/L of each primer, 2.5 mmol/L of each dNTP, and 0.2 U Taq DNA polymerase. The amplification was conducted at 95°C for 4 min followed by 32 cycles at 94°C, 55–60°C for 45 s, and 60 s of elongation at 72°C, with a final extension at 72°C for 8 min. The amplification samples were separated on 8% non-denaturing polyacrylamide gels and the band patterns were visualized by silver staining.

### Powdery mildew evaluation

The alien introgression lines were evaluated for their responses to powdery mildew at the three-leaf stage in a greenhouse at 18/22°C (night/day) with a photoperiod of 14 h of light per day. Ten seeds per line were planted in a single pot (10 cm ×10 cm). NAU0686 seeds were planted as a susceptible control. The local mixture *Bgt* isolates (E09 and E26) were used for inoculation on the seedling leaves. The response of each plant was recorded on a 0–4 IT scale at 10 days post-inoculation when susceptible NAU0686 control plants were heavily diseased. Plants with IT 0–2 were considered resistant and those with IT 3–4 were susceptible (Jin et al., 2021).

In addition, all the V#6 disomic introgression lines and translocation lines were planted in the field nurseries at the Baima Experiment Station of Nanjing Agricultural University for evaluation of powdery mildew responses at the adult-plant stage. Approximately 15 plants were grown in each 1.0-m rows with a 25-cm spacing between rows. Each line was grown in five rows with three replications. The recurrent parent NAU0686 was planted on both sides of each experimental row and served as a susceptible control. A local mixture of *Bgt* isolates (E09 and E26) was used to infect all materials at the jointing stage. Reactions evaluated at the heading stage were recorded on a 0–9 scale with 0–4 considered as resistant and 5–9 considered as susceptible (An et al., 2021).

### Evaluation of agronomic traits

The wheat–*D. villosum* translocation lines NAU4V#6–1, NAU4V#6–2, and NAU4V#6–3 and their recurrent parent NAU0686 were planted in the natural field without powdery mildew disease. Each plot consisted of six rows with each row being 1.5 m long and 0.25 m wide. Twenty seeds per row were hand seeded. Yield trials of each line were performed in a randomized plot design with three replications. The agronomic traits were evaluated in 2022 and 2023 planting seasons. Field management followed local practices. At the physiological maturity stage, 20 plants located in the middle of the internal rows of each material were randomly selected for the analysis of yield-related traits, including plant height, spike length, spike number per plant, seeds per spike, thousand-kernel weight, and grain yield per plant. The mean values and standard errors of the treatments were determined by Microsoft Excel. Differences in agronomic traits between the lines were evaluated by means of Tukey’s *post-hoc* test (SPSS 26.0, USA) at the *p* = 0.05 significance level.

## Results

### Development of a complete set of wheat–*D. villosum* (#6) disomic lines

**Figure 1:**
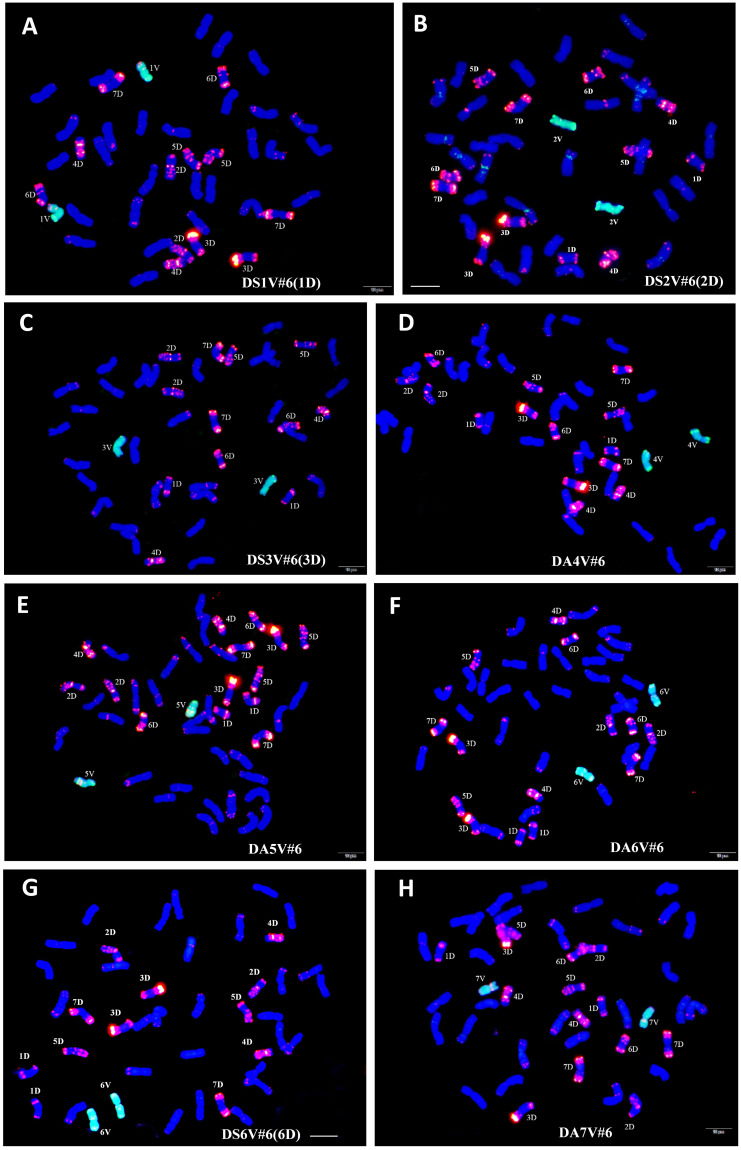
Identification of wheat–*D. villosum* disomic introgression lines through genomic *in situ* hybridization (GISH) and fluorescent *in situ* hybridization (FISH). *D. villosum* 01I139 genomic DNA labeled with fluorescein-12-dUTP (green) as probes was used for GISH. The D-genome-specific probe for FISH was oligo-pAs1–2,5 labeled with TAM (red). Wheat chromosomes were counterstained with DAPI (blue). **(A)** GISH/FISH patterns of NAU1V#6–1 (2n=42), showing a pair of 1D, were substituted by a pair of 1V#6. **(B)** GISH/FISH patterns of NAU2V#6–1 (2n=42), showing a pair of 2D, were substituted by a pair of 2V#6. **(C)** GISH/FISH patterns of NAU3V#6–1 (2n=42), showing a pair of 3D, were substituted by a pair of 3V#6. **(D)** GISH/FISH patterns of NAU4V#6–1 (2n=44), showing a pair of 4V#6, were added in wheat chromosomes. **(E)** GISH/FISH patterns of NAU5V#6–1 (2n=44), showing a pair of 5V#6, were added in wheat chromosomes. **(F)** GISH/FISH patterns of NAU6V#6–1 (2n=44), showing a pair of 6V#6, were added in wheat chromosomes. **(G)** GISH/FISH patterns of NAU6V#6–1 (2n=42), showing a pair of 6D, were substituted by a pair of 6V#6. **(H)** GISH/FISH patterns of NAU7V#6–1 (2n=44), showing a pair of 7V#6, were added in wheat chromosomes. Scale bars, 10 μm.

*D. villosum* accession 01I139 was crossed as a male parent with spring durum cv ZY1286, and their F_1_ plants were subjected to chromosome doubling using colchicine to generate the durum–*D. villosum* amphiploid AABBVV-3 (Hou et al., 2024). Subsequently, AABBVV-3 was crossed with bread wheat cv NAU0686, and backcrossed with NAU0686 three to four times through speed breeding technology. Plants with V chromosomes were firstly identified in the BC_1_F_1_ and BC_2_F_1_ generations using molecular markers that were specific to 14 chromosome arms of *D. villosum* (**Supplementary Table S1**). Furthermore, the alien chromosomes were confirmed by using GISH with a *D. villosum* 01I139 probe and FISH with D genome-specific Oligo-pAs1–2,5 probes. A total of eight fully fertile disomic introgressions were selected in the BC_3_F_2_ or BC_4_F_2_ generations by using molecular cytogenetic technologies (**Figure 1**).

**Figure 2:**
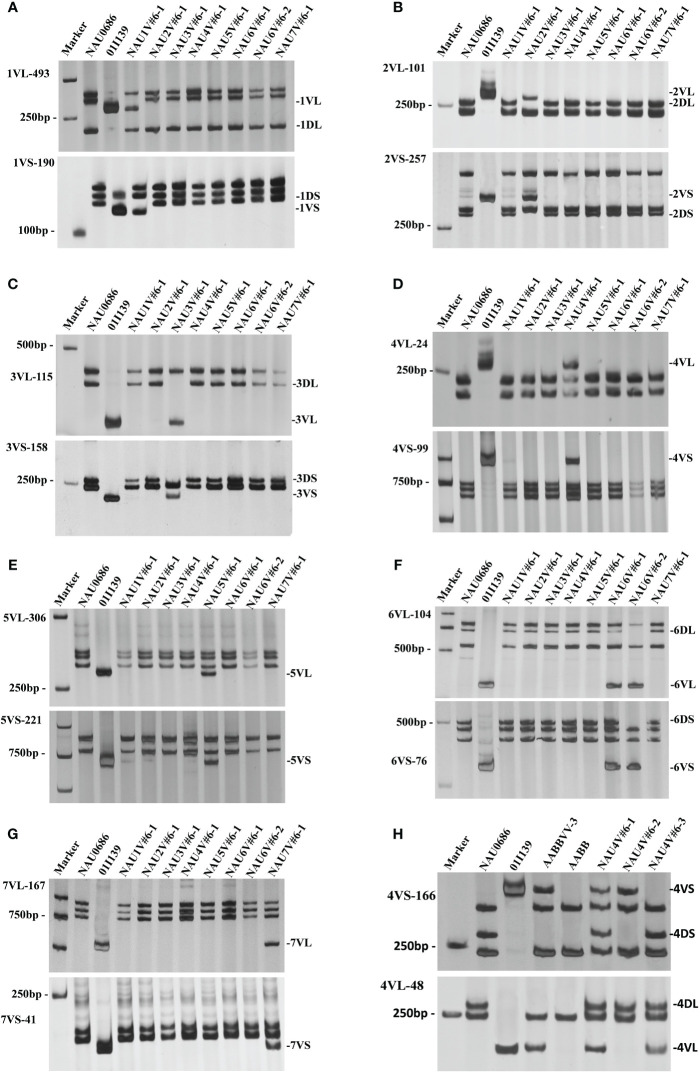
PCR amplification patterns of wheat–*D. villosum* introgression lines using specific molecular markers. **(A)** PCR amplification patterns using 1VL- and 1VS-specific markers 1VL-493 and 1VS-190. **(B)** PCR amplification patterns using 2VL- and 2VS-specific markers 2VL-101 and 2VS-257. **(C)** PCR amplification patterns using 3VL- and 3VS-specific markers 3VL-115 and 3VS-158. **(D)** PCR amplification patterns using 4VL- and 4VS-specific markers 4VL-24 and 4VS-99. **(E)** PCR amplification patterns using 5VL- and 5VS-specific markers 5VL-306 and 5VS-221. **(F)** PCR amplification patterns using 6VL- and 6VS-specific markers 6VL-104 and 6VS-76. **(G)** PCR amplification patterns using 7VL- and 7VS-specific markers 7VL-167 and 7VS-41. **(H)** PCR amplification patterns of 4V#6 translocation lines using 4VS- and 4VL-specific markers 4VL-166 and 4VS-48. Marker, DL2000.

The chromosome component of the eight disomic introgression lines were further identified by the combination of 14 molecular makers and GISH/FISH. These results showed that only the chromosome 1V diagnostic bands of 1VL-493 and 1VS-190 were present in NAU1V#6–1, whereas the chromosome 1D specific bands were absent (**Figure 2A**). The GISH/FISH patterns of NAU1V#6–1 revealed that chromosome 1D was substituted by chromosomes 1V (**Figure 1A**), confirming that NAU1V#6–1 was a DS1V#6 (1D) disomic substitution line. Similarly, chromosome 2V was confirmed to be present and chromosome 2D was confirmed to be absent in NAU2V#6–1 (**Figures 1B**, **2B**), and chromosome 3V was confirmed to be present and chromosome 3D was confirmed to be absent in NAU3V#6–1 using the combination of GISH/FISH and markers (**Figures 1C**, **2C**), suggesting that they were DS2V#6 (2D) and DS3V#6 (3D) disomic substitution lines, respectively. The specific bands of chromosomes 4V and 5V were respectively present in lines NAU4V#6–1 and NAU5V#6–1, but none of the wheat chromosomes were absent in the two lines (**Figures 2D, E**), indicating that NAU4V#6–1 was a DA4V#6 disomic addition line and NAU5V#6–1 was a DA5V#6 disomic addition line (**Figures 1D, E**). Only two lines had the specific bands of chromosome 6V. Of them, NAU6V#6–1 had all the wheat chromosomes, whereas NAU6V#6–2 missed chromosome 6D, suggesting that NAU6V#6–1 was a DA6V#6 disomic addition line (**Figures 1F**, **2F**) and NAU6V#6–2 was a DS6V#6 (6D) disomic substitution line (**Figures 1G**, **2G**). Only the specific bands to chromosome 7V were present in line NAU7V#6–1, but none of the wheat chromosomes were absent (**Figure 2G**), supporting the idea that NAU7V#6–1 was a DA7V#6 disomic addition line, which was also consistent with the cytogenetic result (**Figure 1H**). In summary, a complete set of new wheat–*D. villosum* disomic introgression lines, including four disomic substitution lines (2n=42) that respectively contained chromosomes 1V#6, 2V#6, 3V#6, and 6V#6, and four disomic addition lines (2n=44) that respectively contained chromosomes 4V#6, 5V#6, 6V#6, and 7V#6, were developed.

### Powdery mildew evaluation

**Figure 3:**
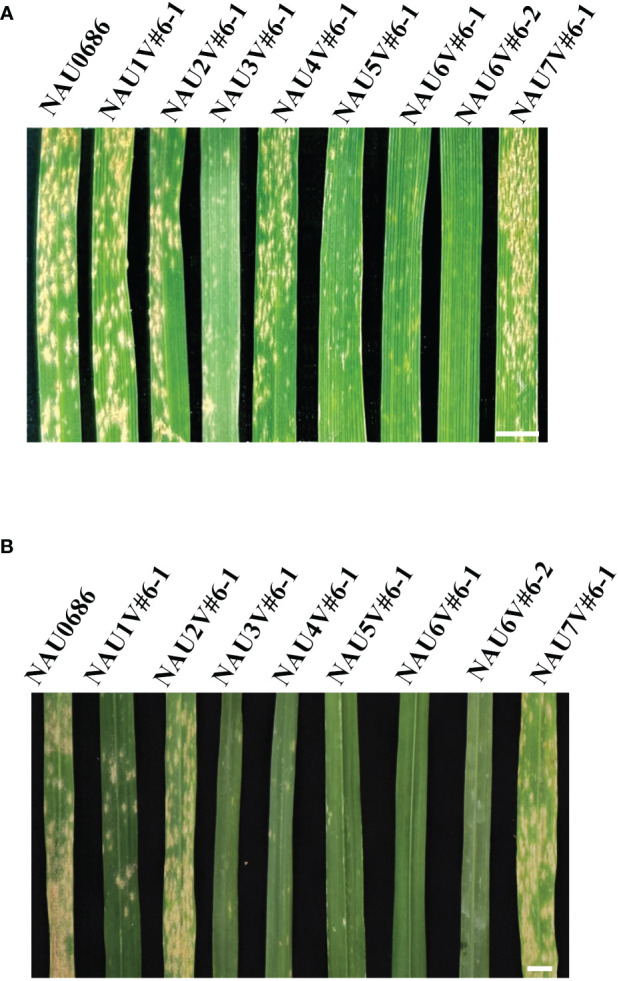
Powdery mildew reactions of a set of wheat–*D. villosum* disomic introgression lines. **(A)** In the seedling (three-leaf) stage, recurrent parent NAU0686, NAU1V#6–1, NAU2V#6–1, NAU4V#6–1, and NAU7V#6–1 were highly susceptible (IT 3–4), and NAU3V#6–1, NAU5V#6–1, NAU6V#6–1, and NAU6V#6–2 were resistant (IT 0–1) to a mixture of *Bgt* isolates. Scale bar, 0.5 cm. **(B)** Lines NAU0686, NAU1V#6–1, NAU2V#6–1, and NAU7V#6–1 were susceptible to powdery mildew at the adult-plant stage, while lines NAU3V#6–1, NAU4V#6–1, NAU5V#6–1, NAU6V#6–1, and NAU6V#6–2 were resistant. Scale bar, 0.5 cm.

To explore the powdery mildew resistance introgressed from *D. villosum* accession 01I139, the eight disomic–introgression lines were assessed for reaction to powdery mildew at both seedling and adult-plant stages. Results showed that four introgression lines NAU3V#6–1, NAU5V#6–1, NAU6V#6–1, and NAU6V#6–2 exhibited resistance to mixture *Bgt* isolates (E09 and E26) at both seedling and adult stages (**Figures 3A, B**), indicating that ASR powdery mildew resistance genes were located on chromosomes 3V#6, 5V#6, and 6V#6. Previously, two genes *Pm55* and *Pm21*, both encoding CNL proteins, were located on short arms of 5V#5 and 6V#4, respectively (Xing et al., 2018; Lu et al., 2024). Thus, the ASR genes on chromosomes 5V#6 and 6V#6 could be allelic to them. In addition, an ASR gene on chromosome arm 3V#6S has been designated as *Pm3VS* (Hou et al., 2024). However, the DA4V#6 line NAU4V#6–1 was susceptible at the seedling stage (IT 4), but highly resistant (IT 1) at the adult-plant stage (IT 1) (**Figure 3B**), suggesting that an APR gene could be located on chromosome 4V#6 of *D. villosum* accession 01I139. The remanent lines of NAU1V#6–1, NAU2V#6–1, and NAU7V#6–1 were all susceptible at both seedling and adult-plant stages. Taken together, a novel powdery mildew APR locus related to chromosome 4V#6 of *D. villosum* accession 01I139 was identified.

### Physical location of the powdery mildew resistant on chromosome 4V#6

**Figure 4:**
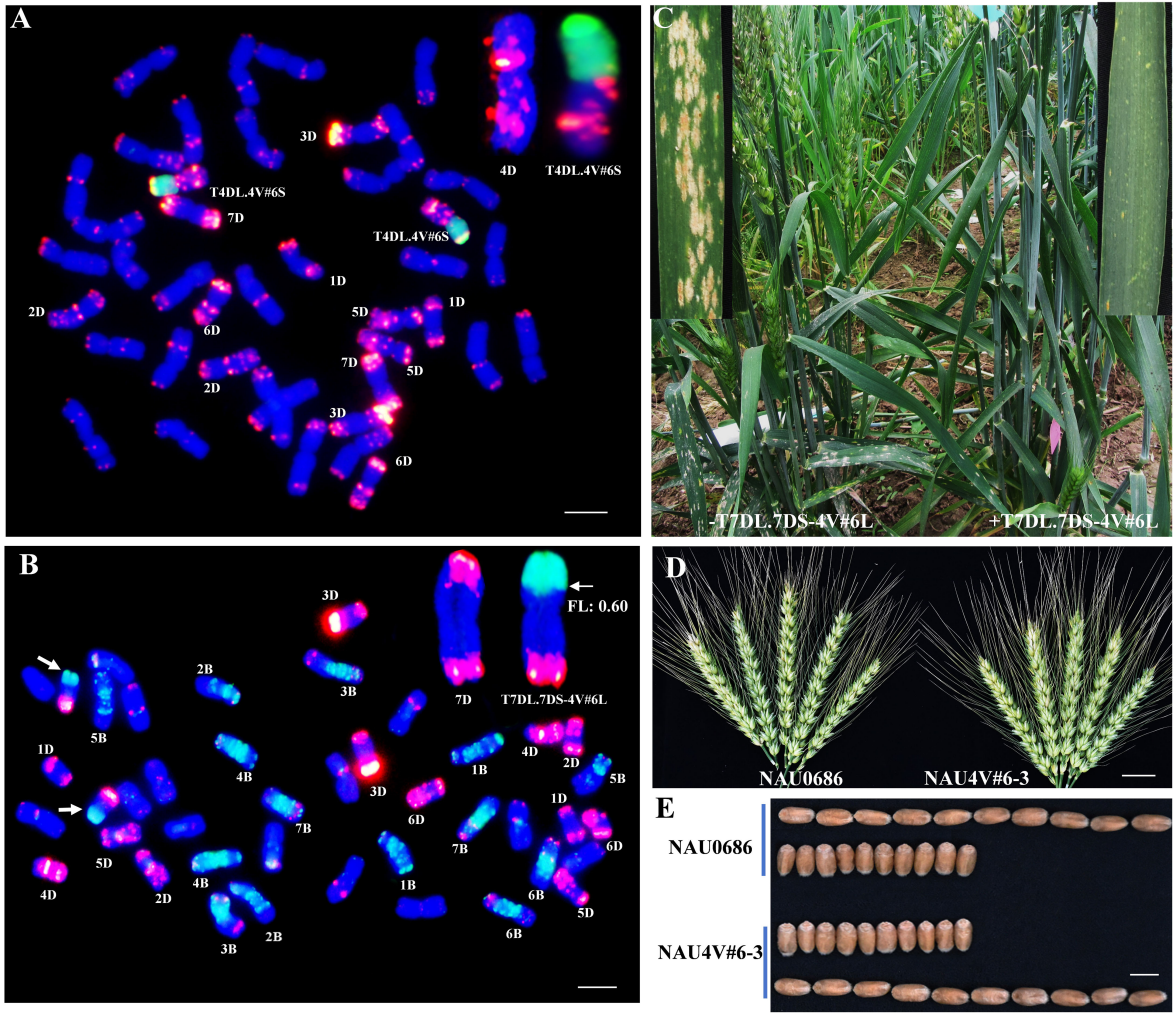
Identification of two wheat–*D. villosum* 4V#6 translocation lines and the plant morphology of the T7DL·7DS-4V#6L line. **(A)** GISH/FISH patterns of NAU4V#6–2 (2n=42), a homozygous T4DL·4V#6S translocation line. **(B)** GISH/FISH patterns of NAU4V#6–3 (2n=42), a homozygous T7DL·7DS-4V#6L translocation line. **(C)** Comparison of powdery mildew response between plant with T7DL·7DS-4V#6L and plant without it in the F_2_ progeny of NAU0686/NAU4V#6–3. **(D)** Comparison of spike length of NAU0686 and T7DL·7DS-4V#6L translocation line NAU4V#6–3. Scale bar, 2.0 cm. **(E)** Comparison of seeds between NAU0686 and T7DL·7DS-4V#6L translocation line NAU4V#6–3. Scale bar, 0.5 cm.

To induce recombination, NAU4V#6–1 was crossed with the *Ph1* gene mutation line NAU0686-ph1b to generate a homozygous *ph1bph1b* population of 27 F_2_ individuals with chromosome 4V#6. Two homologous translocation lines, NAU4V#6–2 and NAU4V#6–3, were identified in the F_4_ progeny of NAU4V#6–1/NAU0686-ph1b. Multicolor FISH (mcFISH) with D- and B-specific genome Oligo-FISH probes and GISH with V genome DNA probes were performed to characterize the recombinant chromosomes. Furthermore, the 4VS-specific molecular marker 4VS-166 and 4VL-specific molecular marker 4VL-48 were used to detect the DNA of NAU4V#6–2 and NAU4V#6–3. Results showed that the band of 4VS was present but the 4DS band was absent, whereas 4VL and 4DL bands were all present in NAU4V#6–3 (**Figure 2H**). Combined with the results of molecular cytogenetic identification, the translocated chromosomes were identified as wheat–*D. villosum* T4DL·4V#6S in NAU4V#6–2 (**Figure 4A**), and T7DL·7DS-4V#6L (**Figure 4B**) in NAU4V#6–3, respectively. Powdery mildew evaluation showed that NAU4V#6–2 was susceptible to a mixture of *Bgt* isolates (E09 and E26) at both seedling and adult-plant stages, whereas NAU4V#6–3 was susceptible at the seedling stage (IT 4) but resistant (IT 1) at the adult-plant stage (**Figure 4C**). These results indicated that the powdery mildew APR locus related to chromosome 4V#6 could be located on the terminal segment of chromosome arm 4V#6L (FL 0.6–1.0).

To confirm whether the resistance was linked to T7DL·7DS-4V#6L, a cross was made between NAU0686 and NAU4V#6–3. All F_1_ seedlings were susceptible to a mixture of *Bgt* isolates (E09 and E26) (IT 4) but showed adult-plant resistance (IT 1–2), similar to NAU4V#6–3. The F_2_ seedlings were firstly examined using a combination of GISH/FISH and the 4VL-specific marker *4VL-48*. Of the 180 seeds identified, 46 individuals lacked alien chromatin, 95 were heterozygous and 39 were disomic for a T7DL·7DS-4V#6L recombinant chromosome pair, suggesting normal gametic transmission of the translocated chromosome relative to its intact chromosome 7D (χ^21:2:1^ = 1.1, *p* > 0.05). These plants were further tested for their reaction to a mixture of *Bgt* isolates at both seedling and adult-plant stages. All of the F_2_ plants were susceptible at the seedling stage. However, the plants with T7DL·7DS-4V#6L translocated chromosome showed high resistance (IT 1–2) at the adult-plant stage, whereas the remaining 46 plants lacking 4V#6L chromatin were still susceptible (IT 7–8) (**Figure 4C**; **Table 2**). As a result, we concluded that a dominant powdery mildew APR gene is located on the alien segment in T7DL·7DS-4V#6L, henceforth designated as *Pm4VL*.

**Table 2:** Responses of F_1_ and F_2_ populations derived from cross NAU0686/NAU4V#6–3 when infected with a mixture of *Bgt* isolates at the adult-plant stage.

Population	No. of infected plants	No. of resistant plants	No. of susceptible plants	Expected ratio	*χ* ^2^	*p-*value
F_1_	30	30	0	–	–	–
F_2_	180	134	46	1:3	0.03	0.14
Infection type (IT)	–	1–2	7–8	–	–	–

### Major agronomic traits investigation of chromosome 4V#6 introgression lines

**Figure 5:**
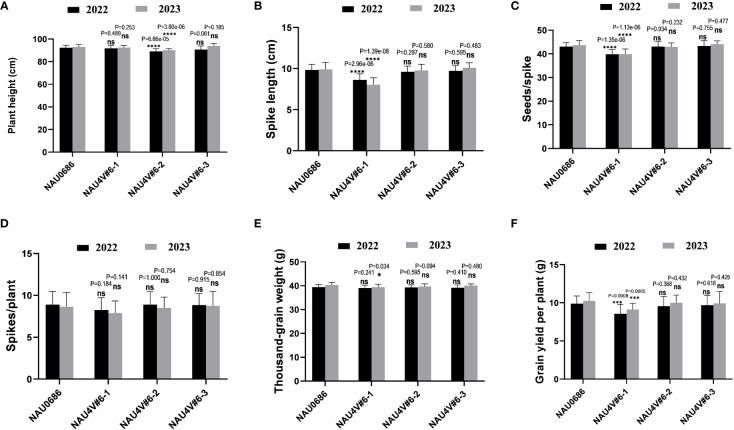
Comparative analysis of agronomic traits between *D. villosum* chromosome 4V#6 introgression lines and recurrent parent NAU0686. **(A–F)** The differences of the major traits in plant height **(A)**, spike length **(B)**, seeds per spike **(C)**, spikes per plant **(D)**, thousand-grain weight **(E)**, and grain yield per plant **(F)** between the 4V#6 introgression lines and their recurrent parent NAU0686. Values are the mean ± SD (two-sided *t*-test, ****p < 0.0001, ***p < 0.001, *P < 0.1; ns, not significant, *n* = 20).

To evaluate the differences in agronomic traits of 4V#6S and 4V#6L translation lines, field traits, including plant height, spike length, spike number per plant, seeds per spike, thousand-kernel weight, and grain yield per plant of NAU4V#6–1, NAU4V#6–2, and NAU4V#6–3, were investigated. All the introgression lines have similar development stages to their background parent NAU0686 under the field conditions in two grown seasons. Statistical analysis revealed no significant differences between the recurrent parent NAU0686 and the T7DL·7DS-4V#6L translation line NAU4V#6–3 in terms of the major traits (**Figures 4C–E**, **5A–F**). However, the DA4V#6 line NAU4V#6–1 exhibited lower spike length, seeds per spike, and grain yield per plant in comparison to NAU0686 plants. In contrast, the T4DL·4V#6S translocation line NAU4V#6–2 showed no significant differences in spike length, spike number per plant, seeds per spike, thousand-kernel weight, and grain yield per plant, but displayed a reduction in plant height of approximately 4.0 cm compared to NAU0686 (**Figure 5A**). These results suggested that T4DL·4V#6S and T7DL·7DS-4V#6L translocations could have no negative impact on wheat yield-related traits.

## Discussion

The narrow genetic base of the current bread wheat varieties hampers efforts to increase yield in a sustainable manner (Zhou et al., 2021). Introgression of natural variations from wild species into bread wheat could contribute to wheat breeding for global food security. *D. villosum* is recognized as a species potentially useful for wheat improvement, as it offers resistance to several important wheat diseases, such as powdery mildew, rust, and sharp eyespot (De Pace et al., 2011). In this study, we developed a complete set of new wheat–*D. villosum* disomic introgression lines from *D. villosum* accession 01I139. All the individual chromosomes of 1V#6 to 7V#6 were identified by GISH/FISH and molecular markers and transferred into a modern wheat cv NAU0686. These disomic addition/substitution lines along with the molecular markers provide the resource bridge to introgress useful variations from the wild species *D. villosum* into the wheat genome using marker-assisted chromosome engineering.

Previously, inoculation of two panels of wheat–*D. villosum* introgression lines with *Bgt* isolate E09 revealed that four chromosome arms, 1VS (*Pm67*), 2VL (*Pm62*), 5VS (*Pm55*/*Pm5V*), and 6VS (*Pm21*), carried genes conferring four types of powdery mildew resistance (Xing et al., 2018; Zhang et al., 2018, 2021, 2022). Among them, the T5DL·5V#4S translocation line with *Pm55* exhibited adult-plant resistance but with susceptible lower leaf sheaths, which is opposite to the response of the T1DL·1V#5S line with *Pm67*, which confers seedling resistance but powdery mildew develops on the leaves of adult plants. *Pm5V*, in a T5DL·5V#5S translocation chromosome, and *Pm21*, in a T6AL·6V#4S translocation chromosome, confer broad-spectrum resistance to powdery mildew at all stages, whereas *Pm62*, in a T2BS·2V#5L translocation chromosome, confers a high resistance to powdery mildew at the adult-plant stage (Zhang et al., 2018). In this study, we evaluated the powdery mildew responses of the third panel of wheat–*D. villosum* introgression lines and showed that three chromosomes, 3V#6, 5V#6, and 6V#6, conferred all-stage resistance to powdery mildew, whereas the chromosome 4V#6 introgression line exhibited resistance to powdery mildew at the adult-plant stage. Through the evaluation of powdery mildew responses of T4DL·4V#6S and T7DL·7DS-4V#6L translocation lines, we further confirmed the presence of an APR gene *Pm4VL* located on the terminal segment (FL 0.6–1.0) of 4V#6L from *D. villosum* accession 01I139. However, the introgression lines of chromosomes 4V#2, 4V#4, and 4V#5 did not confer the resistance to wheat powdery mildew (Zhang et al., 2022). Thus, chromosome 4V-related introgression lines with and without resistance can be used to map-based clone the alien genes through normal recombination on chromosome 4V in wheat background.

Previously, *Pm* gene isolation has shown that introgressed alien genes may be orthologous to resistance genes in wheat, for example, rye-derived *Pm8* homologous to wheat *Pm3* and wheat-derived *Pm12* homologous to *D. villosum Pm21* (Hurni et al., 2013; Zhu et al., 2022). To date, three of the cataloged powdery mildew resistance genes in wheat and its relatives are located on homoeologous group 4 chromosomes, of which *pm61* is a recessive gene located on 4AL-0.8–1.00 (Sun et al., 2018), *Pm66* is an ASR gene on the short chromosome arm of 4S^l^S·4BL from *Aegilops longissima* (Li et al., 2020), and *Pm46/Lr67/Yr46/Sr55* on chromosome arm 4DL confers pleiotropic resistance (Moore et al., 2015). In addition, a wheat–rye line WR41–1 carrying a T4BL·4RL translocated chromosome was resistant to powdery mildew at all stages, indicating that an ASR gene is contained on chromosome arm 4RL of rye (An et al., 2019). The T7DL·7DS-4V#6L translocation line with *Pm4VL* showed powdery mildew resistance at the adult-plant stage that is distinct from the resistance conferred by *pm61*, *Pm66*, *Pm46*, and the ASR gene locus on 4RL. We thus conclude that the APR gene *Pm4VL* could be a novel resistance locus of homoeologous group 4 chromosomes in Triticeae species. Since adult-plant resistance is regarded to be more durable than all-stage resistance, further work to transfer *Pm4VL* to different bread wheat backgrounds would be valuable.

Generally, the translocations induced by *ph1b* mutation are compensating, as the recombination can occur among homologous/homoeologous chromosome segments without the control of *Ph1b* (Türkösi et al., 2022). The T4DL·4V#6S translocation induced by the *ph1b* mutation is expected to produce compensating translocations, but T7DL·7DS-4V#6L appears to be a non-compensating translocation. However, marker analysis using wheat–*D. villosum* deletion lines showed that several markers located on the short arms of CS 7A/7B/7D produced amplicons in DA4V and were physically mapped into the bins of chromosome arm 4VL, suggesting that *D. villosum* chromosome 4V showed some affinity to both wheat homoeologous groups 4 and 7 (Zhang et al., 2017b). Subsequently, the size of the segment translocated from 7VS to 4VL was confirmed by the genome sequencing of *D. villosum* accession 91C43^DH^, which was collinearly larger than that of the fragment translocated from 7BS to 4AL (Zhang et al., 2023). Consequently, the genetic recombination between 4VL and 7DS could be due to the 4VL chromosome segment compensating for the missing fragment of the 7DS wheat chromosome.

Powdery mildew is a serious disease in the main wheat production area of China. The race structure of *Bgt* in China is complex, and only a few known *Pm* genes, such as *Pm21*, *Pm24*, *Pm48*, and *Pm55*, remain effective (Lu et al., 2020; Jin et al., 2022). Accordingly, further novel powdery mildew resistance genes are required to broaden the genetic variability available for use by breeders. The compensatory translocation T7DL·7DS-4V#6L line with APR gene *Pm4VL* may be useful in wheat improvement. Although the effect of this translocated chromosome on yield- and quality-related traits is unknown at present, there were no significant differences in morphological characters between NAU0686 and NAU4V#6–3 when tested in two grown seasons. The plants with T7DL·7DS-4V#6L showed good viability, together with high genetic stability. The similar values obtained for agronomic parameters between NAU0686 and NAU4V#6–3 could be due to the 4VL chromosome segment being homoeologous to the missing fragment of chromosome arm7DS. It is imperative to further evaluate the yield impact of the T7DL·7DS-4V#6L translocated chromosome in various genetic backgrounds in future studies.

## Conclusion

In conclusion, we developed a complete set of new wheat–*D. villosum* disomic introgression lines by molecular cytogenetic methods and found chromosome 4V#6 with a novel adult-plant powdery mildew resistance gene *Pm4VL*. *Pm4VL* was physically located on the terminal segment of 4VL (FL 0.6–1.00). The T7DL·7DS-4V#6L translocation line NAU4V#6–3 with *Pm4VL* showed no obvious negative effect in yield-related traits, providing a new germplasm in breeding for resistance.

## Data availability statement

The original contributions presented in the study are included in the article/**Supplementary material**. Further inquiries can be directed to the corresponding author.

## Author contributions

YW: Data curation, Formal analysis, Visualization, Writing – original draft. TZ: Investigation, Methodology, Software, Writing – original draft. YJ: Investigation, Methodology, Writing – review & editing. WL: Methodology, Software, Writing – review & editing. LK: Data curation, Validation, Writing – review & editing. XL: Investigation, Methodology, Writing – review & editing. LX: Data curation, Methodology, Writing – review & editing. AC: Supervision, Writing – review & editing. RZ: Funding acquisition, Project administration, Resources, Supervision, Writing – review & editing.

## Supplementary material

Supplementary Table 1 PCR-based molecular markers specific to *D. villosum* 1V#6–7V#6 chromosome arms used in the present study.

## References

[B1] An D. G. Han G. H. Wang J. Yan H. W. Zhou Y. L. Cao L. J.. (2021). Cytological and genetic analyses of a wheat-rye 2RL ditelosomic addition line with adult plant resistance to powdery mildew. Crop J. 10, 911–916. doi: 10.1016/j.cj.2021.08.011

[B2] An D. G. Ma P. T. Zheng Q. Fu S. L. Li L. H. Han F. P.. (2019). Development and molecular cytogenetic identification of a new wheat-rye 4R chromosome disomic addition line with resistances to powdery mildew, stripe rust and sharp eyespot. Theor. Appl. Genet. 132, 257–272. doi: 10.1007/s00122-018-3214-3 30374527

[B3] De Pace C. Vaccino P. Cionini G. Pasquini M. Bizzarri M. Qualset C. O. (2011). Wild crop relatives: genomic and breeding resources, cereals, vol 1, chapter 4. Springer, Heidelberg. Exp. Agric 47, 736–736. doi: 10.1017/S0014479711000676

[B4] Develey-Rivière M. P. Galiana E. (2007). Resistance to pathogens and host developmental stage: a multifaceted relationship within the plant kingdom. New Phytol 175, 405–416. doi: 10.1111/j.1469-8137.2007.02130.x 17635216

[B5] Du P. Zhuang L. F. Wang Y. Z. Li Y. Wang Q. Wang D. R.. (2017). Development of oligonucleotides and multiplex probes for quick and accurate identification of wheat and *Thinopyrum bessarabicum* chromosomes. Genome 60, 93–103. doi: 10.1139/gen-2016-0095 27936984

[B6] Friebe B. Jiang J. Raupp W. J. Mclntosh R. A. Gill B. S. (1996). Characterization of wheat–alien translocations conferring resistance to diseases and pets: current status. Euphytica 91, 59–87. doi: 10.1007/BF00035277

[B7] Gao H. Y. Niu J. S. Li S. P. (2018). Impacts of wheat powdery mildew on grain yield and quality and its prevention and control methods. Am. J. Agric. For. 6, 141–147. doi: 10.11648/j.ajaf.20180605.14

[B8] Gupta S. K. Charpe A. Prabhu K. V. Haque Q. M. (2006). Identification and validation of molecular markers linked to the leaf rust resistance gene *Lr19* in wheat. Theor. Appl. Genet. 113, 1027–1036. doi: 10.1007/s00122-006-0362-7 16896713

[B9] Hou F. Chen H. Zhang T. Jin Y. Kong L. Liu X.. (2024). Introgression of an all-stage and broad-spectrum powdery mildew resistance gene *Pm3VS* from *Dasypyrum villosum* chromosome 3V into wheat. Plant Dis. doi: 10.1094/PDIS-11-23-2495-RE 38389384

[B10] Hurni S. Brunner S. Buchmann G. Herren G. Jordan T. Krukowski P.. (2013). Rye *Pm8* and wheat *Pm3* are orthologous genes and show evolutionary conservation of resistance function against powdery mildew. Plant J. 76, 957–969. doi: 10.1111/tpj.12345 24124925

[B11] Jin Y. Liu H. Gu T. Xing L. Han G. Ma P.. (2022). PM2b, a CC-NBS-LRR protein, interacts with TaWRKY76-D to regulate powdery mildew resistance in common wheat. Front. Plant Sci. 13. doi: 10.3389/fpls.2022.973065 PMC9644048 36388562

[B12] Jin Y. L. Shi F. Y. Liu W. H. Fu X. Y. Gu T. T. Han G. H.. (2021). Identification of resistant germplasm and detection of genes for resistance to powdery mildew and leaf rust from 2,978 wheat accessions. Plant Dis. 105, 3900–3908. doi: 10.1094/PDIS-03-21-0532-RE 34129353

[B13] King J. Grewal S. Yang C. Y. Hubbart S. Scholefield D. Ashling S.. (2017). A step change in the transfer of interspecifc variation into wheat from *Amblyopyrum muticum* . Plant Biotechnol. J. 15, 217–226. doi: 10.1111/pbi.12606 27459228 PMC5258861

[B14] Krattinger S. G. Kang J. Bräunlich S. Boni R. Chauhan H. Selter L. L.. (2019). Abscisic acid is a substrate of the ABC transporter encoded by the durable wheat disease resistance gene *Lr34* . New Phytol 223, 853–866. doi: 10.1111/nph.15815 30913300 PMC6618152

[B15] Książczyk T. Apolinarska B. Kulak-Książczyk S. Wiśniewska H. Stojałowski S. Łapiński M. (2011). Identification of the chromosome complement and the spontaneous 1R/1V translocations in allotetraploid *Secale cereale* × *Dasypyrum villosum* hybrids through cytogenetic approaches. J. Appl. Genet. 52, 305–311. doi: 10.1007/s13353-011-0048-y 21584731 PMC3132420

[B16] Li H. Dong Z. Ma C. Xia Q. Tian X. Sehgal S.. (2020). A spontaneous wheat-*Aegilops longissima* translocation carrying *Pm66* confers resistance to powdery mildew. Theor. Appl. Genet. 133, 1149–1159. doi: 10.1007/s00122-020-03538-8 31932954

[B17] Li H. Y. Wei Z. Z. Sela H. Govta L. Klymiuk V. Roychowdhury R.. (2023). Dissection of a rapidly evolving wheat resistance gene cluster by long-read genome sequencing accelerated the cloning of *Pm69* . Plant Commun. 8, 100646. doi: 10.1016/j.xplc.2023.100646 PMC10811346 37415333

[B18] Liu J. Yao Y. Xin M. Peng H. Ni Z. Sun Q. (2022). Shaping polyploid wheat for success: Origins, domestication, and the genetic improvement of agronomic traits. J. Integr. Plant Biol. 64, 536–563. doi: 10.1111/jipb.13210 34962080

[B19] Lu C. Du J. Chen H. Gong S. Jin Y. Meng X.. (2024). Wheat *Pm55* alleles exhibit distinct interactions with an inhibitor to cause different powdery mildew resistance. Nat. Commun. 15, 503. doi: 10.1038/s41467-024-44796-0 38218848 PMC10787760

[B20] Lu P. Guo L. Wang Z. Li B. Li J. Li Y.. (2020). A rare gain of function mutation in a wheat tandem kinase confers resistance to powdery mildew. Nat. Commun. 11, 680. doi: 10.1038/s41467-020-14294-0 32015344 PMC6997164

[B21] McIntosh R. A. Dubcovsky J. Rogers W. J. Xia X. C. Raupp W. J. (2020). Catalogue of gene symbols for wheat: 2020 supplement. Available at: https://wheat.pw.usda.gov/GG3/Wheat_Gene_Catalog_Documents.

[B22] Moore J. W. Herrera-Foessel S. Lan C. Schnippenkoetter W. Ayliffe M. Huerta-Espino J.. (2015). A recently evolved hexose transporter variant confers resistance to multiple pathogens in wheat. Nat. Genet. 47, 1494–1498. doi: 10.1038/ng.3439 26551671

[B23] Qi L. L. Pumphrey M. O. Friebe B. Zhang P. Qian C. Bowden R. L.. (2011). A novel Robertsonian translocation event leads to transfer of a stem rust resistance gene (*Sr52*) effective against race Ug99 from *Dasypyrum villosum* into bread wheat. Theor. Appl. Genet. 123, 159–167. doi: 10.1007/s00122-011-1574-z 21437597

[B24] Sun H. Hu J. Song W. Qiu D. Cui L. Wu P.. (2018). *Pm61*: a recessive gene for resistance to powdery mildew in wheat landrace Xuxusanyuehuang identified by comparative genomics analysis. Theor. Appl. Genet. 131, 2085–2097. doi: 10.1007/s00122-018-3135-1 29967989

[B25] Türkösi E. Ivanizs L. Farkas A. Gaál E. Kruppa K. Kovács P.. (2022). Transfer of the *ph1b* deletion chromosome 5B from Chinese Spring wheat into a winter wheat line and induction of chromosome rearrangements in wheat-*Aegilops biuncialis* hybrids. Front. Plant Sci. 13. doi: 10.3389/fpls.2022.875676 PMC9234525 35769292

[B26] Whalen M. C. (2005). Host defense in a developmental context. Mol. Plant Pathol. 6, 347–360. doi: 10.1111/j.1364-3703.2005.00286.x 20565663

[B27] Xing L. P. Hu P. Liu J. Q. Witek K. Zhou S. Xu J. F.. (2018). *Pm21* from *Haynaldia villosa* encodes a CC-NBS-LRR protein conferring powdery mildew resistance in wheat. Mol. Plant 11, 874–878. doi: 10.1016/j.molp.2018.02.013 29567451

[B28] Zhang R. Yao R. Sun D. Sun B. Feng Y. Zhang W.. (2017b). Development of V chromosome alterations and physical mapping of molecular markers specific to *Dasypyrum villosum.* Mol. Breed 37, 67. doi: 10.1007/s11032-017-0671-3

[B29] Zhang R. Q. Fan Y. L. Kong L. N. Wang Z. J. Wu J. Z. Xing L. P.. (2018). *Pm62*, an adult-plant powdery mildew resistance gene introgressed from *Dasypyrum villosum* chromosome arm 2VL into wheat. Theor. Appl. Genet. 131, 2613–2620. doi: 10.1007/s00122-018-3176-5 30167758

[B30] Zhang R. Q. Lu C. T. Meng X. R. Fan Y. L. Du J. Liu R. R.. (2022). Fine mapping of powdery mildew and stripe rust resistance genes *Pm5V/Yr5V* transferred from *Dasypyrum villosum* into wheat without yield penalty. Theor. Appl. Genet. 135, 3629–3642. doi: 10.1007/s00122-022-04206-9 36038638

[B31] Zhang R. Q. Sun B. X. Chen J. Cao A. Z. Xing L. P. Feng Y. G.. (2016). *Pm55*, a developmental-stage and tissue-specific powdery mildew resistance gene introgressed from *Dasypyrum villosum* into common wheat. Theor. Appl. Genet. 129, 1975–1984. doi: 10.1007/s00122-016-2753-8 27422445

[B32] Zhang R. Q. Xiong C. X. Mu H. Q. Yao R. N. Meng X. R. Kong L. N.. (2021). *Pm67*, a new powdery mildew resistance gene transferred from *Dasypyrum villosum* chromosome 1V to common wheat (*Triticum aestivum* L.). Crop J. 9, 882–888. doi: 10.1016/j.cj.2020.09.012

[B33] Zhang X. Wang H. Sun H. Li Y. Feng Y. Jiao C.. (2023). A chromosome-scale genome assembly of *Dasypyrum villosum* provides insights into its application as a broad-spectrum disease resistance resource for wheat improvement. Mol. Plant 16, 432–451. doi: 10.1016/j.molp.2022.12.021 36587241

[B34] Zhang X. D. Wei X. Xiao J. Yuan C. X. Wu Y. F. Cao A. Z.. (2017a). Whole genome development of intron targeting (IT) markers specifc for *Dasypyrum villosum* chromosomes based on next-generation sequencing technology. Mol. Breed. 37, 115. doi: 10.1007/s11032-017-0710-0

[B35] Zhou Y. Bai S. L. Li H. Su G. L. Zheng D. L. Ma F. F.. (2021). Introgressing the *Aegilops tauschii* genome into wheat as a basis for cereal improvement. Nat. Plants 7, 774–786. doi: 10.1038/s41477-021-00934-w 34045708

[B36] Zhu S. Y. Liu C. Gong S. Chen Z. Chen R. Liu T.. (2022). Orthologous genes *Pm12* and *Pm21* from two wild relatives of wheat show evolutionary conservation but divergent powdery mildew resistance. Plant Commun. 4, 100472. doi: 10.1016/j.xplc.2022.100472 36352792 PMC10030366

