# Supplementary material for: Introgression of an adult-plant powdery mildew resistance gene Pm4VL from Dasypyrum villosum chromosome 4V into bread wheat
Source: Front Plant Sci. 2024 Jun 20;15:1401525. doi: 10.3389/fpls.2024.1401525 (PMC11222578; doi:10.3389/fpls.2024.1401525)
Supplement: Supplementary Table 1 — PCR-based molecular markers specific to D. villosum 1V#6–7V#6 chromosome arms used in the present study. [file Table_1.docx]

| Primer | Chromosome location in CS D genome (bp) | Chromosome location on *D. villosum* V genome (bp) | Primer sequence 5′– 3′ | Tm (℃) |
| --- | --- | --- | --- | --- |
| 1VL-493 | 205284327-205284346 | 225174355 - 225174374 | F: GGCCTTTCATTTGCTTACCA  R: CATGGAGGACCACGAAGTCT | 54 |
|  | 205284819-205284838 | 225174839 - 22517485 8 |  |  |
| 1VS-190 | 88353585-88353604 | 71289316 - 71289335 | F: ACCAGAACTCCAAGCTCTCC  R: CCCTCCGCGTAGATCTTGTA | 56 |
|  | 88353711-88353700 | 71289431 - 71289450 |  |  |
| 2VL-101 | 656385671-656385690 | 635134004 - 635134023 | F: CCGATGGTGAAAGTGCAGTG | 56 |
|  | 656384981-656385000 | 635134216 - 635134235 | R:ATCTCCTCATCCCATCTGCG |  |
| 2VS-257 | 12911785-12911804 | 4755287 - 4755306 | F: CAGACCATGGGAAGAAGGTC | 56 |
|  | 12912134-12912153 | - | R: ACGCTTACCTTGGTTCCTGA |  |
| 3VS-158 | 94951413-94951432 | 85550744 - 85550763 | F: GCTTCTACAGACTTGGCTGC | 56 |
|  | 94951633-94951652 | 85550940 - 85550959 | R: GCCATCTCCACATTAAGCCG |  |
| 3VL-115 | 347302294-347301313 | 483521167 - 483521186 | F: GCTCAAGAGAAGCATTGCCA | 56 |
|  | 347302647-347302668 | 483521433 - 483521454 | R: ACTGCAGTAAAATTTCACCCCT |  |
| 4VL-24 | 230498137-230498156 | 233650497 - 233650478 | F: AGCCTTTCCTGGTGGCATTA | 56 |
|  | 230498325-230498347 | 233650268 - 233650246 | R: GGGAAGATGAGACTTTCTTTGGG |  |
| 4VL-48 | Traes_4DL_4A15E5967.2 |  | F: CTCAGGCTGTCTATAGGTTGTTC | 55 |
|  |  |  | R: GTAGCCCACAAGTCGTTCCA |  |
| 4VS-99 | 1358413-1358432 | - | F: GGGTTTGGGAGCGCAAATAA | 56 |
|  | 1358199-1358218 | - | R: ACCAACCCCTTTCTTCCAGG |  |
| 4VS-166 | Traes_4DS_4836E882E.1 |  | F: GCAAAACTTGTACTTCGTGAACT | 57 |
|  |  |  | R: CCTGTGCCAGCCTTGATTTC |  |
| 5VL-306 | 491064110 -491064128 | 475051993 - 475052011 | F: AGGTGTGGCTAATTCGGACA | 56 |
|  | 491063870-491063888 | 475051730 - 475051749 | R: TCTGCTCCCTACTTAGTGCC |  |
| 5VS-221 | 61894697-61894717 | 40667452 - 40667472 | F: GCAGTGCAGCTAGATATTGGC | 56 |
|  | 61894216-61894237 | 40667962 - 40667983 | R: TGAAACAGATAGGATCCCAGGA |  |
| 6VL-104 | 470660013-470660030 | - | F: GCCGACGCGTTCATTGTC | 56 |
|  | 470660814-470660833 | 550487014 -550487033 | R: TGTTTGCAGACACCGTGTTT |  |
| 6VS-76 | 111224460-111224482 | 95329636 - 95329658 | F: GAGGATATTCTCGCACATGTTTG | 55 |
|  | 111224886-111224905 | 95329907 - 95329924 | R: GCTTGGAGATGTGTCACTGG |  |
| 7VL-167 | 430742765-430742787 | 352691443 - 352691465 | F: CGCAATGCCTAGGAGAAATTTAC | 56 |
|  | 430743282-430743302 | 352691942 - 352691962 | R: GAGCCTTCCAATGCATTCTCA |  |
| 7VS-41 | 102033920-102033937 | 4811016 - 4811033 | F: TGTGCGCCATCCTCCTCC | 56 |
|  | 102033743-102033761 | 4810847 - 4810865 | R: GGTAGCCGAGGATGGTGAG |  |

**Table S1** PCR-based molecular markers specific to *D. villosum* 1V#6-7V#7 chromosome arms used in present study.
